# User characteristics of a smartphone app to reduce alcohol consumption

**DOI:** 10.1007/s13142-017-0477-1

**Published:** 2017-03-17

**Authors:** Claire Garnett, David Crane, Robert West, Susan Michie, Jamie Brown, Adam Winstock

**Affiliations:** 1 Research Department of Clinical, Educational and Health Psychology, University College London, 1-19 Torrington Place, London, WC1E 7HB UK; 2 Cancer Research UK Health Behaviour Research Centre, University College London, 1-19 Torrington Place, London, WC1E 7HB UK; 3 Department of Epidemiology and Public Health, University College London, 1-19 Torrington Place, London, WC1E 7HB UK; 4 Institute of Psychiatry, National Addiction Centre, King’s College London, 4 Windsor Walk, Denmark Hill, London, SE5 8BB UK

**Keywords:** Alcohol, User, Smartphone app, Intervention, Digital

## Abstract

Digital interventions are available to help people reduce their alcohol consumption, but it is not known who uses these interventions and how this treatment-seeking group compares with the general population of drinkers. The study objective was to compare the socio-demographic and drinking characteristics of users of the ‘Drinks Meter’ smartphone app with the general population of drinkers in England and website users of the same intervention. Data were used from the Drinks Meter app and website, and a nationally representative cross-sectional survey in England (Alcohol Toolkit Study). Participants were drinkers aged 16+ in England. Data were collected on participants’ age, gender, region, sexual orientation, social grade and AUDIT score. Regression analyses were conducted to assess differences in socio-demographic and drinking characteristics between samples. Drinks Meter app users, compared with drinkers of the general population, were younger, more likely to be from the South, not heterosexual, less likely to be of a lower social grade and had a higher mean AUDIT score. Drinks Meter app users were younger than website users and reported greater alcohol consumption and related harms. Drinkers using the Drinks Meter app are more likely to be younger and report greater alcohol consumption and related harms compared with the general population of drinkers in England and website users of the same intervention. Apps that provide feedback on drinking appear to be reaching those who report greater alcohol consumption and related harms.

## INTRODUCTION

Approximately 43% of adults worldwide and 88% in western Europe drink alcohol [1]. The global prevalence of alcohol use disorders is 4.9% compared with a prevalence of 6.1% in western Europe [1]. In the UK specifically, excessive alcohol consumption is prevalent with 9.1 million adults drinking at levels above recommended limits [2]. The estimated annual cost to society due to health, social and criminal implications is more than £21 billion [3], and 10.8 million adults in England are at increased risk from their drinking behaviour [4, 5]. An effective strategy for reducing excessive alcohol consumption is to provide brief interventions in primary care settings [6], but less than 10% of excessive drinkers in England report receiving advice on their alcohol consumption from their general practitioner (GP) [7]. A number of widely used digital interventions aimed at helping people cut down on their drinking have been shown to be effective [8, 9]. These digital interventions overcome a number of barriers associated with face-to-face brief interventions as they have low incremental costs, greater reach, avoid the stigma associated with providing or receiving help in person and are highly convenient in that they can be used as and when the individual wants. Smartphone applications (hereon referred to as ‘apps’) have the additional advantage of being available anytime and, therefore, are able to engage people in real time and in their everyday situations. Smart technology is increasingly pervasive; two-thirds of adults in England own a smartphone and over half of all households have a tablet computer [10]. Not only do apps have great potential as a tool for delivering interventions, they can also be used as an assessment tool, being suitable for ecological momentary assessment [11], thereby advancing our understanding of human behaviour and informing future interventions.

Apps for alcohol reduction are already widely available [12], albeit with limited evidence for their effectiveness (based on an early review from 2011 [13] and a recent Cochrane review on digital alcohol interventions [8])﻿; given their potential, it is important to understand the characteristics of people who use the apps. Four apps to reduce alcohol consumption or help patients with alcohol use disorder and that had been evaluated for their efficacy were identified in a state-of-the-art review [14]. Two apps supporting people recovering from alcohol use disorders in the US (‘A-CHESS’ and ‘LBMI-A’) promoted self-reported reductions in alcohol use [15–18], whilst two apps aiming to reduce risky alcohol use amongst Swedish university students (‘Promillekoll’ and ‘PartyPlanner’) failed to promote self-reported reductions in alcohol use [19]. The user characteristics of these four apps were reported: ‘A-CHESS’ app users had a mean age of 38 years, and the majority were male, white and unemployed [15]; ‘LBMI-A’ app users had a mean age of 34 years, about half were male and white, and the majority were employed [16]; and ‘Promillekoll’ and ‘PartyPlanner’ app users had a mean age of 25 and about half were female [19].

Websites are another type of digital intervention, and there are many web-based interventions aimed at excessive drinkers in the general population that have been validated and report the user characteristics [20]. A number of these interventions focus on normative feedback, which typically provides the user with feedback on how their drinking compares with others’ drinking behaviour. These web-based normative feedback interventions were found to have users that consumed excessive levels of alcohol [21–27], had a mean age of around 30 to 40 years old [21–23, 25, 27], had a smaller proportion of female users (ranging from 33 to 47%) [22–25, 27] and had between about one-half and two-thirds of employed users (ranging from 53 to 65%) [21, 23–25]. Whether user characteristics are predictors of a positive treatment outcome was assessed in a web-based intervention that was effective at reducing alcohol consumption at 6-month follow-up in excessive drinkers in the Dutch population [28]. These users had a mean weekly alcohol intake of 44 units and mean age of 46 years, about half were female, and the majority (70%) had high educational backgrounds and considered themselves experienced internet users (78%) [28]. Being female and having higher educational backgrounds were associated with a better treatment response to the web-based intervention at 12-months though there were no user characteristics that were predictors of a positive treatment outcome at the 6-month follow-up [29].

It seems likely that the reported user characteristics for these alcohol reduction websites provide an accurate reflection of those drinkers seeking support from websites as some of these user databases are large, for example 40,000 users of ‘AlcoholScreening.org’ [27]. However, there is a lack of information about who downloads apps to reduce excessive drinking and the potential of apps to deliver effective alcohol reduction interventions is dependent on individuals in need of treatment using the apps, rather than the ‘worried well’. More women and older adults access web-based interventions compared with the population receiving brief interventions [30, 31] but it is unknown how individuals seeking treatment through an app compare with the general population of drinkers or a group seeking web-based treatment. Information about who is accessing these interventions is necessary to tailor materials to excessive drinkers not currently receiving an alcohol reduction intervention.

The socio-demographic characteristics of age, gender, socioeconomic status and region of England tend to be related to alcohol consumption [32]. As people get older, the frequency of drinking tends to increase, but the maximum amount drunk on any 1 day in the last week decreases with age and men tend to be more likely than women to drink at least once a week [32]. Higher income households drink more frequently and the proportion of drinkers varies across regions in England [32]. Sexual orientation is linked with differences in alcohol use among women with non-heterosexual women having a greater prevalence of alcohol use [33] and are more likely to report alcohol-related social consequences [34]. Sexual orientation is linked to fewer differences in alcohol use among men [33, 34]. Younger age groups are also more likely to own a smartphone and to download apps [10]. Mobile phone users in the USA were more likely to use health apps if they were younger, had higher incomes and were more educated [35] though this has not been investigated in alcohol-specific apps or in the UK. Therefore, it is important to assess if users’ socio-demographic characteristics differ between drinkers seeking digital support via an app and the general population of drinkers, and whether any differences are due to socio-demographic differences between the general population who own and use apps and those who do not.

The user characteristics of apps was assessed for ‘Drinks Meter’, a free app that provides the user with instant feedback on their drinking and how the user compares with other users (normative feedback). There are a number of apps relating to alcohol reduction available in the UK [12] though few have transparent intervention content and data available for analysis. The Drinks Meter app has transparent intervention content, data available for analysis and appears popular with users [36].

The Drinks Meter intervention can also be accessed via a website which allows us to compare whether the treatment-seeking groups for apps and websites differ in terms of their socio-demographic and drinking characteristics.

The socio-demographic and drinking characteristics of the general population of drinkers in England was assessed using the Alcohol Toolkit Study (ATS), a national monthly survey tracking alcohol consumption patterns in representative samples of adults in England [37]. The socio-demographic characteristics assessed for both samples were age, gender, social grade, region of England and sexual orientation. The drinking characteristics were based on the Alcohol Use Disorder Identifications Test (AUDIT) questionnaire, a gold-standard measure for assessing alcohol consumption, harmful drinking and alcohol dependence [38].

This paper aims to report the socio-demographic and drinking characteristics of users of the ‘Drinks Meter’ smartphone app and compare them with the general population of (i) drinkers, (ii) drinkers who own a smart digital device with internet access in England and (iii) users of the website version of the ‘Drinks Meter’ intervention.

### Research questions

1. What are the socio-demographic and drinking characteristics of users in England of a popular app (Drinks Meter) to help reduce alcohol consumption?
2. How do these socio-demographic and drinking characteristics compare with the general population in England of (i) drinkers and (ii) drinkers who own a smart digital device with internet access?
3. How do the socio-demographic and drinking characteristics differ between app and website users of Drinks Meter?

## METHODS

### Design

This was an observational study involving anonymised and automated data collection from Drinks Meter users between November 2013 and February 2015. The ATS is a cross-sectional, household, monthly survey of a representative sample of adults in England [37] with data collected between March 2014 and December 2015. The ATS is conducted by Ipsos Mori and uses a type of random location sampling that is a hybrid of random location and quota sampling [37]. England is split into 171,356 areas, each comprising about 300 households, stratified according to a geodemographic analysis of the population [37]. Areas are then randomly allocated to interviewers, who conduct interviews within that area until the quota based on the probability of being at home is fulfilled. This sampling method is often considered superior to conventional quota sampling [37] and has been shown to result in a sample that is nationally representative in its socio-demographic composition [39].

### Study sample

Participants were included in the analysis if they met the following criteria: aged 16 and over, lived in England and had complete cases (9.0% of Drinks Meter data and .9% of ATS data had missing cases). Participants who reported ‘never’ to the question ‘How often do you have a drink containing alcohol?’ were excluded. This resulted in a total of 27,358 participants from the different samples—818 users of the Drinks Meter app, 24,299 from the ATS (11,990 who reported owning a smart digital device with internet access) and 2241 users of the Drinks Meter website.

### Measures

Socio-demographic characteristics of age (in years), gender (male/female), social grade (ABC1/C2DE), region in England (North/South) and sexual orientation (heterosexual/not heterosexual) were measured. Social grade in the ATS was assessed using the National Readership Survey social-grades system (ABC1 = higher and intermediate professional/managerial, and supervisory, clerical, junior managerial/administrative/professional and C2DE = skilled, semi-skilled, unskilled manual and lowest grade workers or unemployed) [40]. Data on social grade from the Drinks Meter intervention were derived from occupation into ABC1 or C2DE classifications. Region in England was defined by government office region (North = North East, North West, Yorkshire and the Humber, East Midlands and West Midlands; and South = London, South East, South West and East of England). Sexual orientation was assessed by asking participants to self-identify as heterosexual, bisexual, homosexual or prefer not to say. These responses were then dichotomised into heterosexual and not heterosexual (bisexual, homosexual and prefer not to say).

Drinking characteristics of participants were based on the Alcohol Use Disorders Identification Test (AUDIT) questionnaire. This 10-item questionnaire assesses alcohol consumption, alcohol dependence and harmful drinking. The possible scores range from 0 to 40 and are categorised into four different zones indicating lower-risk drinking (0–7), hazardous drinking (8–15), harmful drinking (16–19) and at-risk of alcohol dependence (20–40). The AUDIT -Consumption (AUDIT-C) questionnaire consists of the first three-items of the full questionnaire. The AUDIT-C assesses alcohol consumption and possible scores range from 0 to 12. For the current study, higher risk consumption was indicated by an AUDIT-C score ≥5. The AUDIT-C is used as a brief screening tool for alcohol misuse in primary care and a cut-off of ≥5 has improved specificity [41, 42]. The binge drinking measure was based on AUDIT question 3: ‘how often do you have 6 or more standard drinks on one occasion?’ The possible responses of ‘never’, ‘less than monthly’, ‘monthly’, ‘weekly’, ‘daily’ or ‘almost daily’ were dichotomised into ‘less than monthly’ or ‘monthly or more’ to represent whether the individual was a regular binge-drinker, in line with the definition used in the National Survey on Drug Use and Health in the US [43]. Whether others had expressed concern with regard to their drinking was based on AUDIT question 10: ‘has a relative or friend or a doctor or another health worker ever been concerned about your drinking or suggested you cut down?’ The possible responses of ‘no’, ‘yes, but not in the last 6 months’ and ‘yes, during the last 6 months’ were dichotomised into ‘yes’ or ‘no’.

The ATS assessed whether participants owned a smart digital device with internet access (participants answered yes to owning a ‘web-enabled mobile or smart phone’ or ‘tablet’ and ‘access to internet: via a mobile terminal’).

### Intervention

The Drinks Meter app is freely available on iTunes and Google Play Store. On downloading the app, users enter socio-demographic and drinking data. The app provides instant personalised feedback on the user’s drinking (weekly units and calories based on their drinking data) and normative feedback on how their drinking compares with other Drinks Meter users. Users then complete a ‘risk adjuster’ questionnaire and are provided with the same feedback adjusted for their personal risk factors (based on personal and family medical history, if drugs are consumed whilst drinking, and pregnancy). Users then complete the AUDIT questionnaire and receive feedback on their level of alcohol-related risk (including health risks) and some suggestions on what to do based on the reported level of risk. The app follows a tunnelled approach so all users receive the same intervention content. Only 100 users (of 818) responded to questions reviewing the app. Of the 12.2% who responded, the majority would recommend the Drinks Meter app to friends and family (94.0%; n=94) and found it easy to use (91.0%; n=91). Over half of the 100 users responding to questions reported planning to drink less after using the Drinks Meter app (58.0%; n=58) and the re-maining users (42.0%; n=42) reported no plan to change their drinking rather than drinking more. The Drinks Meter website is also freely accessible (http://www.drinksmeter.com) and follows the same intervention approach as for the app.

### Analysis

Descriptive statistics were used to report the socio-demographic and drinking characteristics of the Drinks Meter app and website users, and participants in the ATS: drinkers in the general population, and drinkers in the general population who owned a smart digital device with internet access. Data from Drinks Meter were unweighted. Data from the ATS were weighted to match an English population profile [37] to provide an accurate comparison of the population prevalence for the Drinks Meter data. Regression analyses were conducted to assess differences in socio-demographic and drinking characteristics between the Drinks Meter app users and ATS samples and Drinks Meter website users. Univariable and multivariable linear regressions were conducted for continuous dependent variables (age, AUDIT score, AUDIT-C score) and logistic regressions for binary dependent variables (gender, region, sexual orientation, social grade, binge drinking, and others concerned). Issues of collinearity arose between the drinking variables. Therefore, in the multivariable models, for each socio-demographic characteristic, the other socio-demographic variables and AUDIT score were included as covariates, and for each drinking characteristic, only the socio-demographic variables were included as covariates.

## RESULTS

### What are the socio-demographic and drinking characteristics of users in England of a popular app (Drinks Meter) to help reduce alcohol consumption?

Table 1 reports the socio-demographic and drinking characteristics of Drinks Meter users. The mean age was 30.6 and the majority of users were male (64.9%), of a high social grade (92.8%), from the South of England (71.1%) and heterosexual (83.5%). Drinks Meter users had a mean AUDIT score of 12.3, a mean AUDIT-C score of 6.3 and 63.9% took part in binge drinking at least monthly. The majority of users had not had a relative, friend, doctor or health worker who expressed concern with regard to their drinking (70.0%).

**Table 1:** Socio-demographic and drinking characteristics of Drinks Meter app users and drinkers in the general population

Variable	Drinks Meter app users *N* = 818	Drinkers *N* = 24,299	*B* or OR (95% CI) *p* value	Adjusted *B* or OR (95% CI)^a^ *p* value
Socio-demographic characteristics				
Gender, % (95% CI)			OR = .58 (.50, .67) *p* < .001	OR = 1.17 (1.00, 1.38) *p* = .054
Male^b^	64.9 (61.6, 68.2)	51.7 (51.1, 52.3)		
Female	35.1 (31.8, 38.4)	48.3 (47.7, 48.9)		
Age in years, mean (SD)	30.6 (11.9)	47.8 (18.3)	*B* = −17.23 (−18.49, −15.97) *p* < .001	*B* = −10.78 (−12.08, −9.47) *p* < .001
Region of England, % (95% CI)			OR = 2.18 (1.87, 2.54) *p* < .001	OR = 2.89 (2.45, 3.40) *p* < .001
North^b^	28.9 (25.8, 32.0)	46.9 (46.3, 47.5)		
South	71.1 (68.0, 74.2)	53.1 (52.5, 53.7)		
Sexual orientation, % (95% CI)			OR = 2.79 (2.30, 3.38) *p* < .001	OR = 2.27 (1.83, 2.81) *p* < .001
Heterosexual^b^	83.5 (81.0, 86.0)	93.4 (93.1, 93.7)		
Not heterosexual	16.5 (14.0, 19.0)	6.6 (6.3, 6.9)		
Social grade, % (95% CI)			OR = .12 (.09, .15) *p* < .001	OR = .12 (.09, .16) *p* < .001
ABC1^b^	92.8 (91.0, 94.6)	60.2 (59.6, 60.8)		
C2DE	7.2 (5.4, 9.0)	39.8 (39.2, 40.4)		
Drinking characteristics				
AUDIT score, mean (SD)	12.3 (6.3)	4.9 (3.8)	*B* = 7.38 (7.11, 7.66) *p* < .001	*B* = 6.46 (6.20, 6.73) *p* < .001
AUDIT-C score, mean (SD)	6.3 (2.0)	4.1 (2.5)	*B* = 2.17 (1.99, 2.34) *p* < .001	*B* = 1.64 (1.47, 1.81) *p* < .001
Binge drinking, % (95% CI)			OR = 6.12 (5.29, 7.08) *p* < .001	OR = 4.18 (3.58, 4.88) *p* < .001
Less than monthly^b^	36.1 (32.8, 39.4)	77.5 (77.0, 78.0)		
Monthly or more	63.9 (60.6, 67.2)	22.5 (22.0, 23.0)		
Others concerned, % (95% CI)			OR = 8.61 (7.34, 10.11) *p* < .001	OR = 7.58 (6.36, 9.05) *p* < .001
No^b^	70.0 (66.9, 73.1)	95.3 (95.0, 95.6)		
Yes	30.0 (26.9, 33.1)	4.7 (4.4, 5.0)		

*AUDIT* Alcohol Use Disorders Identification Test, *OR* odds ratio, *B* regression coefficient

^a^Drinking variables adjusted for socio-demographic variables only; socio-demographic variables adjusted for other socio-demographic variables and AUDIT score

^b^Reference group

### How do these socio-demographic and drinking characteristics compare with that of the general population of (i) drinkers in England?

Table 1 reports the results from the regression analyses. Both in the univariable and multivariable regressions, Drinks Meter users were significantly younger (*B*_adj_ = −10.78, *p* < .001), more likely to be from the South of England (OR_adj_ = 2.89, *p* < .001), not heterosexual (OR_adj_ = 2.27, *p* < .001) and less likely to be of a lower social grade (OR_adj_ = .12, *p* < .001) than the general population of drinkers. Drinks Meter users were less likely to be female than the general population of drinkers (OR_adj_ = .58, *p* < .001), but after adjusting for other socio-demographic characteristics and AUDIT score, there was no significant difference between the samples (OR_adj_ = 1.17, *p* = .054). Both in the univariable and multivariable regressions, Drinks Meter users had significantly higher mean AUDIT (*B*_adj_ = 6.46, *p* < .001) and AUDIT-C scores (*B*_adj_ = 1.64, *p* < .001), and they were more likely to binge drink monthly or more (OR_adj_ = 4.18, *p* < .001), and have others who expressed concerns regarding their drinking (OR_adj_ = 7.58, *p* < .001) than drinkers in the general population.

### How do these socio-demographic and drinking characteristics compare with that of the general population of (ii) drinkers who owned a smart digital device with internet access in England?

Table 2 reports the results comparing Drinks Meter users with drinkers of the general population who owned a smart digital device with internet access. The pattern of results remained the same as in the comparison with all drinkers. Drinks Meter users were younger than drinkers of the general population who owned a smart digital device with internet access though the difference was not as large in this comparison.

**Table 2:** Socio-demographic and drinking characteristics of Drinks Meter app users and drinkers in the general population who owned a smart digital device with internet access

Variable	Drinks Meter app users *N* = 818	Drinkers who own a smart device with internet access *N* = 11,990	*B* or OR (95% CI) *p* value	Adjusted *B* or OR (95% CI)^a^ *p* value
Socio-demographic characteristics				
Gender, % (95% CI)			OR = .60 (.52, .70) *p* < .001	OR = 1.08 (.91, 1.27) *p* = .393
Male^b^	64.9 (61.6, 68.2)	52.7 (51.9, 53.6)		
Female	35.1 (31.8, 38.4)	47.3 (46.5, 48.2)		
Age in years, mean (SD)	30.6 (11.9)	40.2 (14.7)	*B* = −9.63 (−10.66, −8.61) *p* < .001	*B* = −7.93 (−9.02, −6.83) *p* < .001
Region of England, % (95% CI)			OR = 2.32 (1.98, 2.71) *p* < .001	OR = 3.07 (2.58, 3.64) *p* < .001
North^b^	28.9 (25.8, 32.0)	48.4 (47.6, 49.3)		
South	71.1 (68.0, 74.2)	51.6 (50.8, 52.5)		
Sexual orientation, % (95% CI)			OR = 3.32 (2.72, 4.05) *p* < .001	OR = 2.37 (1.87, 2.99) *p* < .001
Heterosexual^b^	83.5 (81.0, 86.0)	94.4 (94.0, 94.8)		
Not heterosexual	16.5 (14.0, 19.0)	5.6 (5.2, 6.0)		
Social grade, % (95% CI)			OR = .14 (.11, .19) *p* < .001	OR = .13 (.10, .17) *p* < .001
ABC1^b^	92.8 (91.0, 94.6)	64.8 (64.0, 65.6)		
C2DE	7.2 (5.4, 9.0)	35.2 (34.4, 36.0)		
Drinking characteristics				
AUDIT score, mean (SD)	12.3 (6.3)	5.4 (4.0)	*B* = 6.85 (6.56, 7.14) *p* < .001	*B* = 6.40 (6.11, 6.70) *p* < .001
AUDIT-C score, mean (SD)	6.3 (2.0)	4.5 (2.5)	*B* = 1.82 (1.64, 1.99) *p* < .001	*B* = 1.63 (1.45, 1.80) *p* < .001
Binge drinking, % (95% CI)			OR = 4.54 (3.91, 5.26) *p* < .001	OR = 3.86 (3.30, 4.52) *p* < .001
Less than monthly^b^	36.1 (32.8, 39.4)	71.9 (71.1, 72.7)		
Monthly or more	63.9 (60.6, 67.2)	28.1 (27.3, 28.9)		
Others concerned, % (95% CI)			OR = 7.71 (6.52, 9.12) *p* < .001	OR = 7.98 (6.61, 9.62) *p* < .001
No^b^	70.0 (66.9, 73.1)	94.7 (94.3, 95.1)		
Yes	30.0 (26.9, 33.1)	5.3 (4.9, 5.7)		

*AUDIT* Alcohol Use Disorders Identification Test, *OR* odds ratio, *B* regression coefficient

^a^Drinking variables adjusted for socio-demographic variables only; socio-demographic variables adjusted for other socio-demographic variables and AUDIT score

^b^Reference group

### How do the socio-demographic and drinking characteristics differ between app and website users of Drinks Meter?

Table 3 reports the results comparing app and website users of the Drinks Meter intervention. Drinks Meter app and website users did not differ in terms of gender (OR_adj_ = .87, *p* = .110), region of England (OR_adj_ = .90, *p* = .225), sexual orientation (OR_adj_ = 1.08, *p* = .503), or social grade (OR_adj_ = .85, *p* = .306) though the app users were significantly younger than website users (*B*_adj_ = −3.42, *p* < .001). App users’ AUDIT (*B*_adj_ = 1.16, *p* < .001) and AUDIT-C (*B*_adj_ = .21, *p* = .021) score were both significantly higher than website users. App users were more likely to take part in binge drinking monthly or more (OR_adj_ = 1.20, *p* = .033) and have others concerned with regards to their drinking (OR_adj_ = 1.37, *p* = .001).

**Table 3:** Socio-demographic and drinking characteristics of Drinks Meter app users and website users

Variable	Drinks Meter app users *N* = 818	Drinks Meter website users *N* = 2241	*B* or OR (95% CI) *p* value	Adjusted *B* or OR (95% CI)^a^ *p* value
Socio-demographic characteristics				
Gender, % (95% CI)			OR = .87 (.73, 1.02) *p* = .088	OR = .87 (.73, 1.03) *p* = .110
Male^b^	64.9 (61.6, 68.2)	61.5 (59.5, 63.5)		
Female	35.1 (31.8, 38.4)	38.5 (36.5, 40.5)		
Age in years, mean (SD)	30.6 (11.9)	34.6 (12.3)	*B* = −3.99 (−4.97, −3.01) *p* < .001	*B* = −3.42 (−4.36, −2.47) *p* < .001
Region of England, % (95% CI)			OR = .92 (.77, 1.10) *p* = .346	OR = .90 (.75, 1.08) *p* = .255
North^b^	28.9 (25.8, 32.0)	27.1 (25.3, 28.9)		
South	71.1 (68.0, 74.2)	72.9 (71.1, 74.7)		
Sexual orientation, % (95% CI)			OR = 1.11 (.90, 1.38) *p* = .336	OR = 1.08 (.86, 1.35) *p* = .503
Heterosexual^b^	83.5 (81.0, 86.0)	84.9 (83.4, 86.4)		
Not heterosexual	16.5 (14.0, 19.0)	15.1 (13.6, 16.6)		
Social grade, % (95% CI)			OR = .76 (.57, 1.03) *p* = .079	OR = .85 (.62, 1.17) *p* = .306
ABC1^b^	92.8 (91.0, 94.6)	90.8 (89.6, 92.0)		
C2DE	7.2 (5.4, 9.0)	9.2 (8.0, 10.4)		
Drinking characteristics				
AUDIT score, mean (SD)	12.3 (6.3)	10.8 (6.6)	*B* = 1.44 (.92, 1.96) *p* < .001	*B* = 1.16 (.64, 1.67) *p* < .001
AUDIT-C score, mean (SD)	6.3 (2.0)	6.0 (2.3)	*B* = .27 (.09, .44) *p* = .003	*B* = .21 (.03, .38) *p* = .021
Binge drinking, % (95% CI)			OR = 1.35 (1.15, 1.59) *p* < .001	OR = 1.20 (1.02, 1.43) *p* = .033
Less than monthly^b^	36.1 (32.8, 39.4)	43.2 (41.2, 45.3)		
Monthly or more	63.9 (60.6, 67.2)	56.8 (54.8, 58.9)		
Others concerned, % (95% CI)			OR = 1.28 (1.07, 1.53) *p* = .007	OR = 1.37 (1.14, 1.64) *p* = .001
No^b^	70.0 (66.9, 73.1)	74.9 (73.1, 76.7)		
Yes	30.0 (26.9, 33.1)	25.1 (23.3, 26.9)		

*AUDIT* Alcohol Use Disorders Identification Test, *OR* odds ratio, *B* regression coefficient

^a^Drinking variables adjusted for socio-demographic variables only; socio-demographic variables adjusted for other socio-demographic variables and AUDIT score

^b^Reference group

## Discussion

The majority of users of an app to help reduce alcohol consumption were relatively young, male, of a high social grade, from the South of England and heterosexual. The mean AUDIT score of the Drinks Meter app users was 12.3 and the mean AUDIT-C score was 6.3 indicating hazardous drinking and higher risk consumption. The majority of Drinks Meter app users took part in binge drinking at least once a month. When adjusting for other character- istics, Drinks Meter app users were significantly youn- ger than the general population of drinkers, and more likely to be of a higher social grade, from the South of England and not het- erosexual. There was no significant difference in gender after adjustment for other socio-demographic characteristics and AUDIT score. Drinks Meter app users compared with the general population of drinkers report greater alcohol consumption and more alcohol-related harms. This pattern of results, for socio-demographic and drinking characteristics, was the same when comparing Drinks Meter app users against only those drinkers who were digitally engaged though the age gap between groups was smaller in this comparison. These users who accessed the Drinks Meter through the app were more likely to be younger and report greater alco-hol consumption and harms than those who accessed the intervention via the website. There-fore, individuals in need of support, not the ‘wor-ried well’, are using alcohol reduction apps.

These results suggest that alcohol reduction apps reach people with a wide range of demographic characteristics though some groups of the general population of drinkers (those who are older, from the North of England and of a lower social grade) are less likely to use an alcohol reduction app. Smartphone ownership is more likely among younger age groups [10] and people of a higher socioeconomic status [44] though the differences in socio-demographic and drinking characteristics between app users and the general population remained when selecting only those who owned a smart digital device with internet access. This suggests that it is not simply ownership of a suitable device that is driving differences in demographic characteristics between users of the Drinks Meter app and the general population of drinkers. This is an important point to consider in terms of providing interventions equitably to excessive drinkers. One possible way to address this is to ensure that in health care settings with brief advice, GPs know that certain groups of drinkers may need additional prompting as they are less likely to search for and use an alcohol reduction app. Another is for local areas to promote digital interventions as part of their approach to reducing alcohol related harm. Such an initiative has just commenced in Lancashire in the North of England who are utilising a bespoke version of the Drinks Meter app for identification and brief advice (IBA) across the region.

A major strength of this study is that it is the first to assess the user characteristics of an alcohol reduction app, and to compare them with the general population of drinkers and website users of the same intervention. The characteristics of patients using an app (A-CHESS) for support in recovery from alcoholism were assessed and it was found that the majority of patients were male, white and unemployed with a mean age of 38 years [15]. These patients were enrolled into a study that involved using this app, so whilst these findings are interesting, they do not indicate who in the general population of drinkers are seeking help with reducing their alcohol consumption. In web-based normative feedback interventions, users in the general population had a mean age of 40 [21–23,25], less than half were women [22–25] and over half were employed [21, 23–25]. Users of these interventions tended to consume excessive levels of alcohol [21–26]. Drinks Meter users were excessive drinkers and the majority were male, which is consistent with the findings from the web-based normative feedback interventions. However, Drinks Meter users had a mean age of 31, younger than most of the mean ages for users of the web-based interventions. Another strength of this study is that the Drinks Meter users were not enrolled or recruited into a trial but found the app on iTunes or the Google Play Store. Therefore, the Drinks Meter users are representative of individuals who want to reduce their alcohol consumption and seek digital support, which mirrors the real-world situation for most users of behaviour change apps.

The user characteristics of a smoking cessation app (SmokeFree28) have been assessed and compared with smokers in the general population who were trying to stop; users were more likely to be younger, female, have a non-manual occupation and higher daily cigarette consumption [45]. Although the proportion with a non-manual occupation was higher than in the general population of smokers trying to stop, the difference was small and the authors note that social gradient in app usage merits further investigation [45]. The socio-demographic characteristics of the Drinks Meter users were a younger, more affluent group, even when comparing the users with the general population who own a smart digital device with access to the internet. Unlike in users of SmokeFree28, there was no difference in gender after adjusting for other factors between Drinks Meter users and the general population of drinkers in England. The findings from these studies suggest that users of apps for changing health behaviours tend to be more dependent, younger, and of a higher social grade than the general population. However, there is not much data available and these findings need to be validated with other health behaviour change apps.

This study has limitations. It relied on data from a single app and so these findings are not necessarily generalizable to users of other alcohol reduction apps. The Drinks Meter app focuses on providing feedback on behaviour and normative feedback, but it does not include a number of other intervention techniques known to be used frequently in other popular alcohol reduction apps such as facilitating self-monitoring [12]. Some apps use daily self-monitoring to promote alco- hol reduction though none of these [13], or any other alcohol reduction apps, including Drinks Meter, have been empirically tested. The 12.2% referred to here are indeed a convenience sample that is likely to be more motivated and engaged in the app. However, the data reported in the results is not a convenience sample but from all app users regardless of their engagement with the app. Future research should assess the extent to which these results generalise to other alcohol reduction apps, ideally those that have been empirically validated. This highlights the importance of more developers providing open-access data so that this field of research can advance more rapidly and results can be validated amongst a number of alcohol reduction apps. Another limitation of this study was that the measure for social grade was not identical between samples. The Drinks Meter intervention also had no measure as to whether the users were receiving any formal treatment for their drinking or whether they were included in the Alcohol Toolkit Study.

To conclude, drinkers seeking digital support through an app compared with the general population of drinkers in England report greater alcohol consumption and related harms, including more frequent binge drinking. These drinkers were also more likely to be younger, of a higher social grade, from the South of England, and not heterosexual. These differences still existed when the general population of drinkers was selected for owning a smart digital device with internet access. Drinkers using an app-based intervention were more likely to be younger and report greater alcohol consumption and harms than those using the same intervention through a website. This suggests that users of these apps for alcohol reduction are not the ‘worried well’ but reaching those who report greater alcohol consumption and related harms. This is the first study to report the characteristics of treatment-seeking users of an alcohol reduction app for the general population of drinkers. The Drinks Meter users were treatment-seeking individuals, rather than being enrolled or recruited into a trial, and so this sample is likely to be representative of most users of behaviour change apps in the real-world. These findings need to be validated with other alcohol reduction apps and if these findings are generalizable, then health care providers should be aware that particular groups may need more prompting to seek digital help.
